# Steroidogenic Factor 1 (*Nr5a1*) is Required for Sertoli Cell Survival Post Sex Determination

**DOI:** 10.1038/s41598-019-41051-1

**Published:** 2019-03-14

**Authors:** Prashanth Anamthathmakula, Chandra Suma Johnson Miryala, Rebecca S. Moreci, Chandrashekara Kyathanahalli, Sonia S. Hassan, Jennifer C. Condon, Pancharatnam Jeyasuria

**Affiliations:** 1 Department of Obstetrics and Gynecology, Wayne State University Perinatal Initiative, School of Medicine, Wayne State University, Mott Center for Growth and Development, Detroit, MI USA; 2 Department of Cell Biology, Duke University, Durham, NC 27708 USA

## Abstract

The elevated level of Steroidogenic Factor 1 (*Nr5a1, Sf-1*) expression in the male gonadal development pathway, post sex determination, implies a vital role in testis gonadal differentiation. In this study we generated Sertoli cell-specific *Nr5a1* KO mice (SC-SF-1^−/−^) at E14.5, which coincides with testis development post sex determination, using the *Amh-Cre* mouse model. Analysis of SC-SF-1^−/−^ (Sertoli cell specific *Nr5a1* knockout) testes demonstrated apoptosis as early as E15. Further analysis revealed that SC-SF-1^−/−^ gonads displayed lower MDM2 levels resulting in elevated TP53 levels, which we believe may lead to apoptosis of the Sertoli cell population, inferring the possibility that NR5A1 directly regulates MDM2 expression. By E15.5, the Sertoli cell and germ cell population declined in SC-SF-1^−/−^ mice resulting in the disruption of seminiferous cords with limited cord structure remaining at E18.5. Due to the loss of Sertoli and germ cells, the testis weights of SC-SF-1^−/−^ mice at 6-weeks were much reduced; however, SC-SF-1^−/−^ seminal vesicles weights were comparable suggesting intact Leydig cell androgen production. We conclude that NR5A1 regulates the TP53 pathway during development, is essential for fetal Sertoli cell survival and controls the cell cycle of Sertoli cells during differentiation.

## Introduction

Steroidogenic factor 1 (NR5A1), an orphan nuclear receptor was initially discovered as a transcription factor that regulated enzymes and cholesterol transport proteins in the steroidogenic pathway^1,2^. The *Nr5a1* null mice demonstrated added functions that included adrenal, gonadal, pituitary and ventromedial hypothalamic developmental programs^3,4^. Conditional NR5A1 knockouts of the pituitary, ventromedial hypothalamus and Leydig cells in the developing gonad added significant knowledge to the function of this nuclear receptor^5–8^. The complete loss of function of NR5A1 in the null mouse results in dysgenesis of the gonadal and adrenal primordia through apoptosis by E11.5 soon after these NR5A1 positive tissue precursors separate to become their prospective organs^9^. The mechanism through which this apoptosis occurs is unknown. Gonadal dysgenesis is not seen in heterozygous *Nr5a1* null mutation in the mouse whereas heterozygous mutations of *NR5A1* in humans may result in both gonadal dysfunction and dysgenesis (streak gonads)^10^. This discrepancy may be accounted for by the presence of a functional allele in the mouse whereas in human mutations the expressed mutant allele may have dominant negative effects on development. It is of interest that disorders of sex development due to mutations of *NR5A1* in humans are rarely associated with adrenal dysfunction^10^ suggesting that many mutations of *NR5A1* do not affect steroidogenesis but affect pathways associated with the gonadal development. The Sertoli cell is the initial cell in the testis to functionally differentiate at E11.5 in mouse gonadal development following initiation of the male developmental pathway and *Sry* (sex-determining region Y) expression. SRY together with NR5A1 upregulate *Sox9* (Sry-Box 9) expression by binding the TES sequence (testis specific enhancer of Sox9) on the *Sox9* promoter^11^. In the differentiating Sertoli cells, SOX9 and NR5A1 then bind the promoter of anti mullerian hormone (*Amh*) and upregulate *Amh* expression^12^. The function of the Sertoli cell in the developing testis is to form seminiferous cords, cause Mullerian derivative degeneration, prevent meiosis in germ cells and direct fetal Leydig cell function/development^13^. AMH expression is only seen in the fetal testis and not the fetal ovary during the prenatal period, it is expressed in females in granulosa cells after primary follicle recruitment^14–16^ and is used as a marker for ovarian reserve for *in vitro* fertilization (IVF) in women of advanced age^17^.

The expression profile of NR5A1 in male human embryonic gonads parallels that of the mouse prior to and post gonadal differentiation^18^. In the male mouse *Nr5a1* is first expressed at the urogenital ridge at E9.5 and thereafter continues in the Leydig cells and Sertoli cells throughout postnatal and adult life. *Nr5a1* is down regulated in the ovary after sex determination at E11.5 while the continued expression of *Nr5a1* after *Sry* expression in the XY gonad is coupled to its role for male differentiation^19^. The complete loss of *Nr5a1* in null mutants results in gonadal dysgenesis in both males and females and this occurs in the bi-potential gonad after the gonadal and adrenal primordia separate, between E9.5 and E11.5 prior to sex determination^4^. The dysgenesis of the gonad in *Nr5a1* null mice precludes functional studies of NR5A1 in differentiation as well as function in the adult gonad. In a previous study we generated a conditional knockout of *Nr5a1* at E11.5 in the fetal Leydig cells using the *Amhr2-Cre* mice in order to overcome these limitations. The ablation of *Nr5a1* caused a proliferation deficit phenotype in fetal Leydig cells while steroidogenesis and testosterone synthesis was markedly curtailed resulting in cryptorchid testes and the loss of androgen dependent structures (seminal vesicles, epididymis etc.)^7^. We did not study this mouse model past early development because we believe that there was a mixed Sertoli cell/ Leydig cell phenotype due to Cre expression in both cell types post development. Kato *et al*.^20^ however used the *Amhr2-Cre* model to study *Nr5a1* ablation in the Sertoli cells and concluded that NR5A1 was essential for maturation and spermatogenesis in postnatal testes. In order to understand the developmental and cellular functions of NR5A1 in Sertoli cells of developing testis post sex determination, we developed a Sertoli cell specific knockout of *Nr5a1* using the previously well-defined *Amh-Cre* mouse model. The Cre recombinase is expressed at E14.5^21,22^ when *Sry* expression and the male differentiation pathway have already been established. In this study, we conditionally deleted the *Nr5a1* gene in Sertoli cells and demonstrate that NR5A1 is required for the maintenance and survival of developing Sertoli cells. The evidence from this study infers that *Nr5a1* ablation results in Sertoli cell loss by apoptosis and leads to the eventual loss of primordial germ cells (PGCs). Furthermore, we have evidence that *Nr5a1* ablation induced apoptosis is mediated through the MDM2/TP53 pathway. This is the first study to demonstrate the role of NR5A1 as a possible survival and cell cycle checkpoint in the embryonic stages of male sexual development.

## Results

### Generation of a Sertoli cell-specific conditional *Nr5a1* knockout mouse line

In the present study, we generated a Sertoli cell-specific *Nr5a1* knockout mouse line (*Nr5a1*^*fl/fl*^ *Amh-Cre*, hereafter referred to as SC-SF-1^−/−^; while *Nr5a1*^*fl/fl*^ (served as control) to establish the function of *Nr5a1* in Sertoli cells during testicular development and spermatogenesis. Adult *Nr5a1*^*fl/fl*^ females carrying the Cre recombinase driven from the Amh promoter are fertile. Therefore our mating strategy included female *Nr5a1*^*fl/fl*^ *Amh-Cre* mice mated to male *Nr5a1*^*fl/fl*^ mice to generate SC-SF-1^−/−^ male fetuses. The *Amh-Cre*^23–26^ and *Nr5a1*^*fl/fl*^ mice^5–7^ have been used extensively. In previous studies^21,27^ the Sertoli cell specificity of the Amh-Cre mouse was shown using the *R2R* reporter mice. The specificity of this model for Sertoli cell expression has been used to purify Sertoli cells from embryonic testis using FAC sorting^28^. To determine the extent and specificity of Cre mediated recombination, we performed immunofluorescence for NR5A1 in E15.5 control and SC-SF-1^−/−^ testes (Supplementary Fig. 1). The cords are well formed in the control testis at E15.5. In the SC-SF-1^−/−^ mice there was a loss of testis cord structure compared to control.

### Reduced expression of SOX9 and loss of proliferation in the Sertoli cell population of SC-SF-1^−/−^ testes

**Figure 1:**
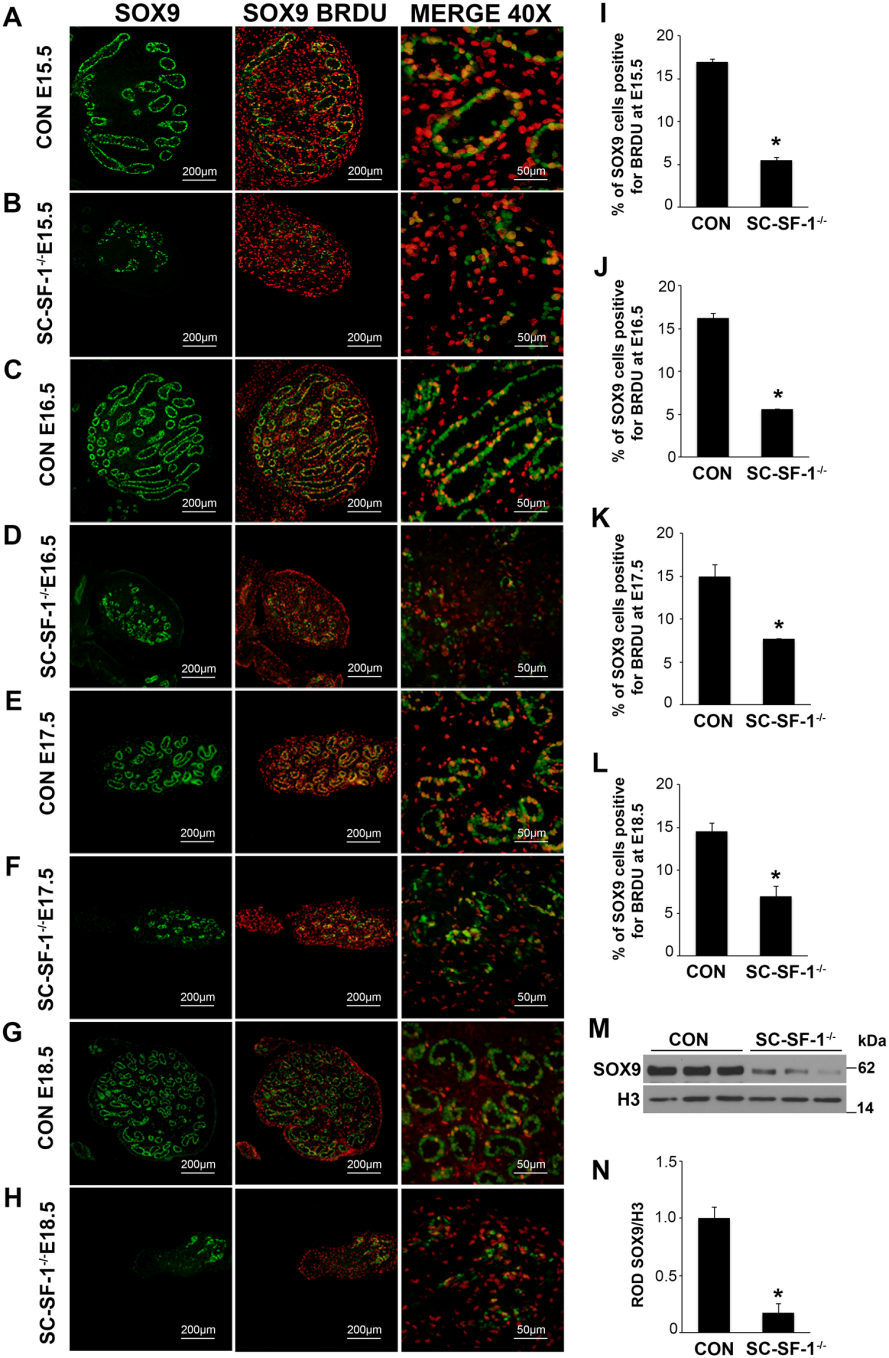
Reduced expression of SOX9 and loss of proliferation of Sertoli cells in SC-SF-1^−/−^ mice. Immunostaining of SOX9 and BrdU in developing testes of control and SC-SF-1^−/−^ mice at E15.5 (**A**,**B**), E16.5 (**C**,**D**), E17.5 (**E**,**F**) and E18.5 (**G**,**H**). SC-SF-1^−/−^ testes demonstrated a marked decline in SOX9 positive cells (**B**,**D**,**F**,**H**) compared to control testes (scale bar = 200 μm). Pregnant mice were injected with BrdU (50 mg/kg b.wt.) 4 h prior to sacrifice. Reduced BrdU incorporation was observed in SC-SF-1^−/−^ testes (**B**,**D**,**F**,**H**) compared to controls (**A**,**C**,**E**,**G**). Proliferating Sertoli cells in the seminiferous tubules were identified with SOX9 and BrdU double positive staining (orange; merge 40X). Reduced proliferating Sertoli cells were observed in SC-SF-1^−/−^ testes through E15.5 to E18.5 (**I**–**L**) compared to their respective controls. (**M**) SOX9 protein levels were analyzed by western blotting in whole tissue extracts of control and SC-SF-1^−/−^ mice at E15.5. Histone H3 served as a protein loading control. (**N**) Quantification of western blots demonstrated marked decline in SOX9 expression in SC-SF-1^−/−^ testes compared to control testes. *p < 0.001 by two-tailed Student’s unpaired *t* test. CON = control.

SOX9, a marker for Sertoli cells, is highly upregulated at E11.5 in the embryonic Sertoli cells of the developing male testes following *Sry* expression and testis determination^29,30^. Utilizing immunohistochemistry we analyzed SOX9 expression in SC-SF-1^−/−^ testes to determine whether Sertoli cell specific ablation of *Nr5a1* would decline SOX9 levels. There was an immediate loss of SOX9 positive cells in the testes of SC-SF-1^−/−^ mice (Fig. 1B,D,F,H) compared to control testes (Fig. 1A,C,E,G) at E15.5 through E18.5. We observed a loss of 75% of Sertoli cells at E15.5 and this was maintained at 81% by E18.5 in the SC-SF-1^−/−^ testes compared to their controls (Supplementary Fig. 1B). The immediate loss of SOX9 positive cells in SC-SF-1^−/−^ testes strongly suggested an apoptotic phenotype but also prompted us to the question whether there was a loss of proliferation in Sertoli cells. Utilizing BrdU incorporation as a measure of proliferation, we observed a general reduction in BrdU staining in E15.5 SC-SF-1^−/−^ testes demonstrating a definitive loss of proliferation especially in the Sertoli cell population when compared to the controls. As shown in Fig. 1B, the Sertoli cells in the SC-SF-1^−/−^ testes have lost their ability to proliferate as seen in the merge between SOX9 and BrdU staining (orange cells). A similar result was also observed in the E16.5 and E18.5 SC-SF-1^−/−^ testes (Fig. 1D,F,H). In our analysis, we found that the proportion of proliferating Sertoli cells is significantly lower in E15.5 through E18.5 SC-SF-1^−/−^ testes compared to their respective controls (Fig. 1I–L). We also examined SOX9 expression in SC-SF-1^−/−^ fetal testes at E15.5 by immunoblotting and observed a significant drop (*p* < 0.001) in SOX9 levels when compared to the control testis (Fig. 1M,N). Based on these results our findings strongly suggest that NR5A1 is required for continued maintenance of SOX9 expression in development post sex determination.

### The loss of *Nr5a1* in Sertoli cells may result in apoptosis in the SC-SF-1^−/−^ testis

**Figure 2:**
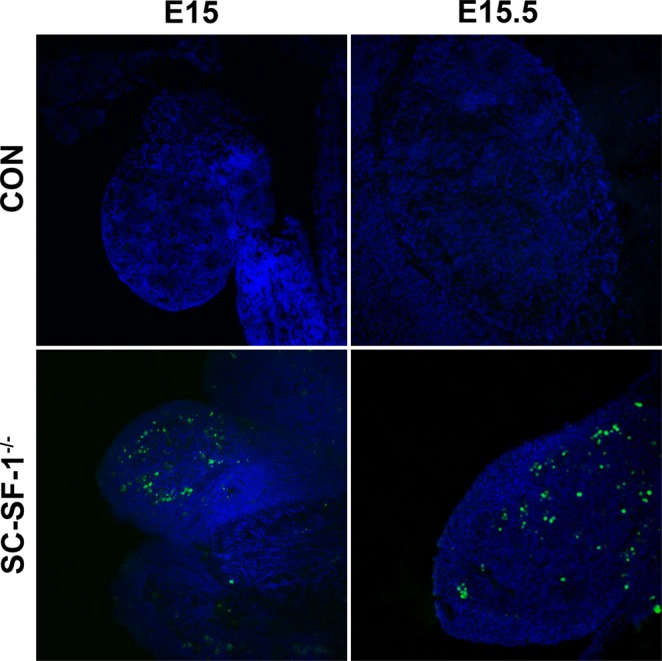
Apoptosis in Sertoli cell specific NR5A1 knockout testes. TUNEL assay was performed with testes at E15 and E15.5 from control and SC-SF-1^−/−^ embryos to detect apoptotic cells. TUNEL-positive cells were detected using confocal fluorescence microscopy and images were taken at 10X magnification (scale bar = 200 μm). Apoptotic cells were observed in the SC-SF-1^−/−^ testes, but not in the control mice. CON = control. The nuclei are counterstained with DAPI (blue).

The loss of cords in the SC-SF-1^−/−^ testes suggests that Sertoli cells may have undergone apoptosis. Therefore we performed TUNEL staining on E15–18.5 testes from 3 control and 3 SC-SF-1^−/−^ mice. Figure 2 illustrates numerous apoptotic cells in SC-SF-1^−/−^ embryos as early as E15, 12 hrs post *Nr5a1* ablation in the Sertoli cells, and continued to E15.5. No TUNEL positive cells were observed in controls or in the SC-SF-1^−/−^ testes from embryonic stages E16.5, 17.5 and 18.5 (data not shown). These data suggest that *Nr5a1* ablation at E14.5 caused an immediate and early apoptosis phenotype. The loss of the Sertoli cell population and increase in apoptotic indices as indicated by increased TUNEL staining suggests Sertoli cell apoptosis may occur upon *Nr5a1* ablation and contributes to the loss of seminiferous cords in the testes of SC-SF-1^−/−^ mice.

### *Nr5a1* ablation leads to the loss of MDM2 in Sertoli cells of testes resulting in TP53-mediated apoptosis

**Figure 3:**
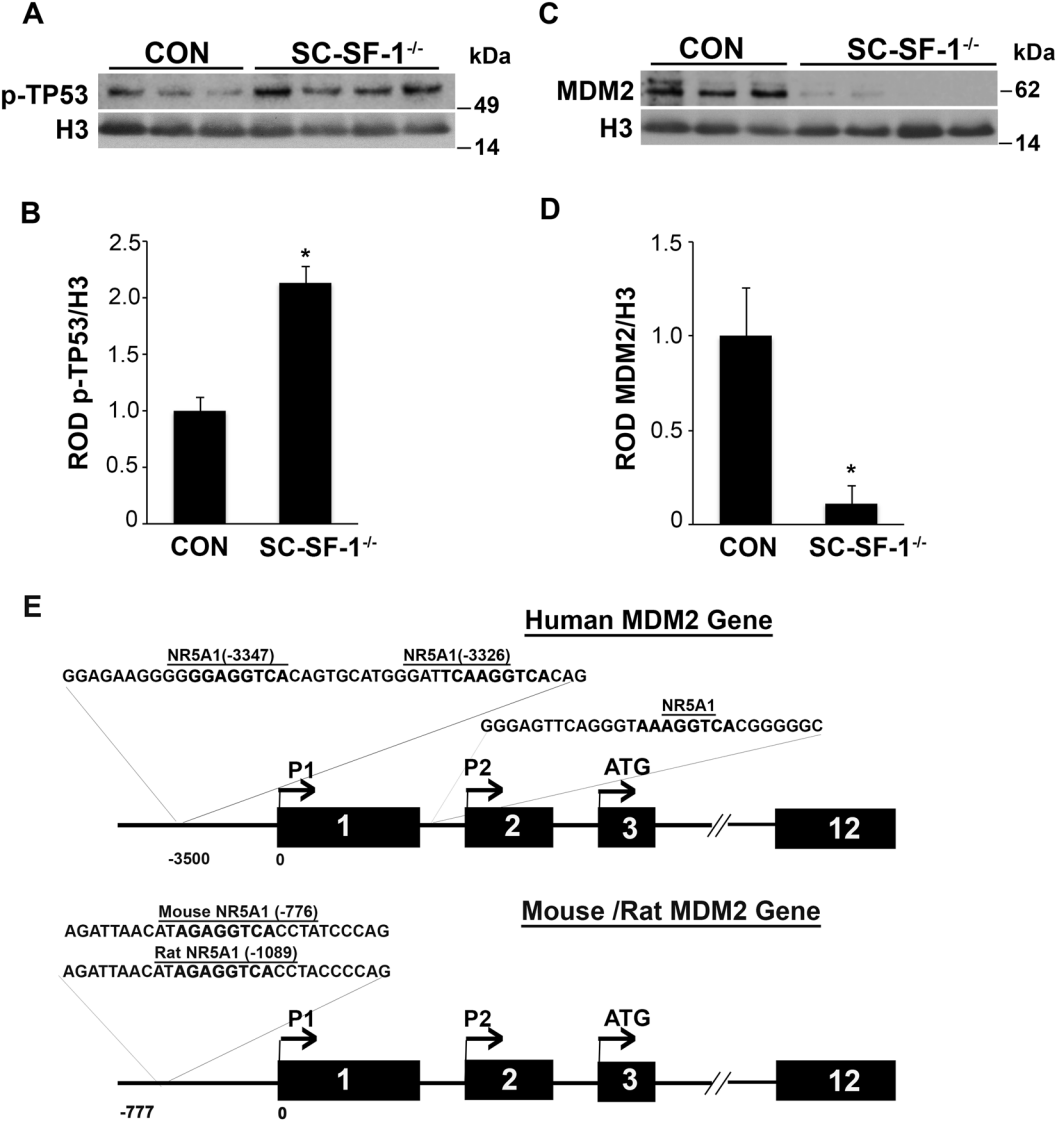
Expression of phospho-TP53 (p-TP53) and MDM2 in SC-SF-1^−/−^ mice. The activation of TP53 was investigated in testis of SC-SF-1^−/−^ mice. (**A**) Western blot and (**B**) densitometric analysis of p-TP53 (Ser15) in whole tissue extracts of control and SC-SF-1^−/−^ mice at E15. The expression of p-TP53 levels was higher in SC-SF-1^−/−^ testes. (**C**) MDM2 protein levels were analyzed by western blotting. (**D**) Densitometric analysis of MDM2 showing a decrease in the protein level. The protein levels were normalized and plotted against Histone H3. *p < 0.01 by two-tailed Student’s unpaired *t* test. (**E**) Sequence analyses of 5′ UTR of MDM2 gene in human, rat and mouse species. Human *MDM2* gene displaying multiple SF-1 response elements in the P1 (5′ to exon1) and P2 (in intron1) promoter region. Rat and mouse *Mdm2* gene have an NR5A1 response element in a highly conserved region in the P1 promoter at position −1089 and −776 respectively upstream of transcription start site. NR5A1 response element sequence is depicted in bold. CON = control.

*Nr5a1* ablation induced apoptosis in SC-SF-1^−/−^ testes as early as E15. We investigated the role of TP53 in this process. TP53 functions to maintain genomic integrity by transcriptional activation of genes involved in cell cycle arrest, DNA repair, or apoptosis in response to cellular damage. Phosphorylation of TP53 at serine 15 is required to activate the apoptotic cascade. We isolated total lysates from E15 testis and observed increased levels of p-TP53 (Ser15) in SC-SF-1^−/−^ testes, as demonstrated by western blot analysis (Fig. 3A,B; *p* < 0.01). TP53 is negatively regulated by murine double minute 2 (MDM2). Western blot analysis revealed very low levels of MDM2 protein in testes lysate of SC-SF-1^−/−^ embryos compared to the controls (Fig. 3C,D; *p* < 0.01). We next analyzed the *Mdm2* gene for potential NR5A1 binding sites using Clustal W sequence alignment algorithm (MacVector software). Figure 3E demonstrates the map with NR5A1 response element sequences in human, rat and mouse species. Sequence analyses of human *MDM2* gene revealed the presence of multiple NR5A1 response elements in the P1 and P2 promoter region. We also found an NR5A1 response element in a highly conserved region in the P1 promoter of both rat (−1089 position) and mouse (−776 position) *Mdm2* gene. The sequence alignment is detailed in Supplementary Fig. 3.

### A strong reduction in AMH expression in SC-SF-1^−/−^ Sertoli cells

**Figure 4:**
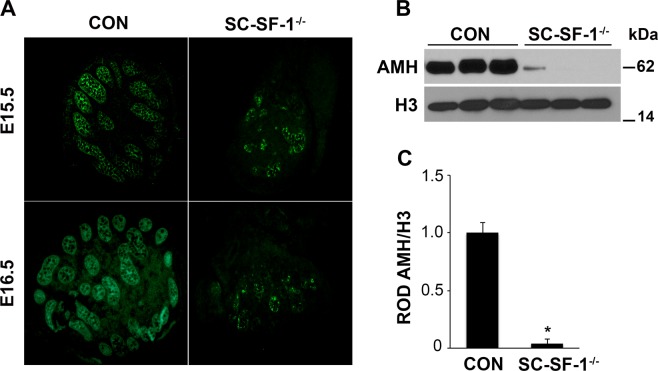
Loss of NR5A1 in Sertoli cells decreases AMH expression. (**A**) Immunostaining of AMH in developing testes of control and SC-SF-1^−/−^ mice. Dissected testes from E15.5 and E16.5 demonstrated a marked decline in AMH expression as well as a loss of seminiferous cords in the SC-SF-1^−/−^ embryos. (**B**) AMH protein levels were analyzed by western blotting in whole tissue extracts of control and SC-SF-1^−/−^ mice at E15.5. Histone H3 served as a protein loading control. (**C**) Quantification of western blots demonstrated marked decline in AMH expression in SC-SF-1^−/−^ testes compared to control testes. *p < 0.001 by two-tailed Student’s unpaired *t* test. CON = control.

To determine *Nr5a1* role in the regulation of AMH in Sertoli cells, we used both immunohistochemistry and immunoblot analysis to analyze AMH levels in testes of SC-SF-1^−/−^ embryos. Immunohistochemistry revealed decreased AMH staining in E15.5 and E16.5 testes in SC-SF-1^−/−^ embryos when compared to control testes (Fig. 4A). Further, immunoblot analysis using whole testes demonstrated a significant (*p* < 0.001) loss of AMH expression at E15.5 in the SC-SF-1^−/−^ testis compared to control (Fig. 4B). Densitometric analysis revealed a ∼10 fold reduction in AMH protein levels in the E15.5 SC-SF-1^−/−^ testes (Fig. 4C).

### Sertoli cell specific ablation of *Nr5a1* results in germ cell loss

**Figure 5:**
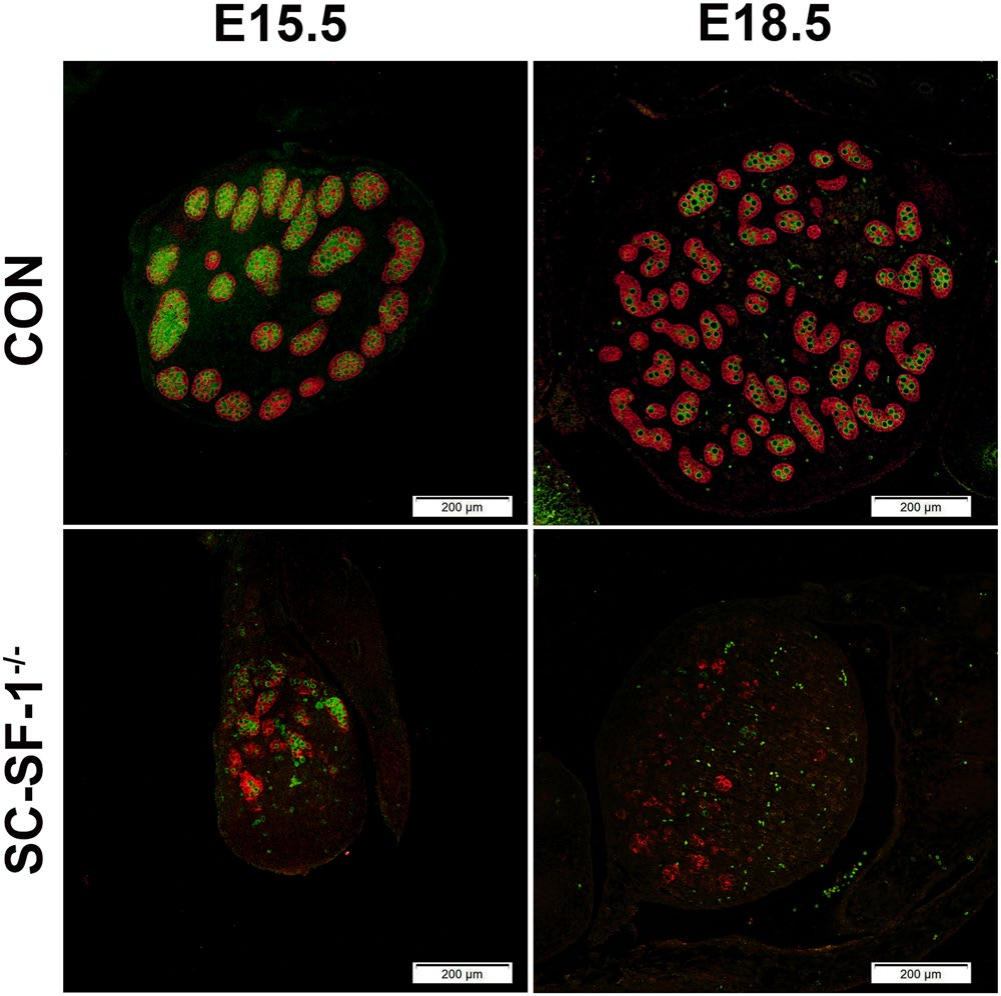
*Nr5a1* ablation in Sertoli cells led to gradual loss of germ cells. Double immunostaining for AMH and VASA was performed in testes at E15.5 and E18.5 from control and SC-SF-1^−/−^ embryos. Sertoli cells labeled by AMH and germ cells labeled by VASA depict a normal cord-like structure in the control testes. A pronounced loss of AMH and VASA staining was observed in SC-SF-1^−/−^ testes suggesting a marked decline in Sertoli cell and germ cell population (scale bar = 200 μm). CON = control.

Germ cells are reliant on Sertoli cells for nutritional and immunological support and would die in their absence. To elucidate the fate of germ cells following *Nr5a1* ablation, germ cell populations were analyzed in developing SC-SF-1^−/−^ testes. Germ cells were identified using VASA staining and Sertoli cells by AMH immunostaining in testis sections from E15.5 to E18.5. As expected, there was a significant loss of germ cells at E15.5 (Fig. 5), probably due to the loss of the germ cell niche normally afforded to them by Sertoli cells as observed in the control testis. This loss continued through E16.5 to E17.5 resulting in very few germ cells (Supplementary Fig. 2) and by E18.5 the testes sections were mostly devoid of germ cells in SC-SF-1^−/−^ embryos. The green fluorescence in E18.5 SC-SF-1^−/−^ testes was mostly auto fluorescence or non-specific fluorescence. Some AMH immunoreactivity is still seen in E18.5 SC-SF-1^−/−^ testes and would represent Sertoli cells that escaped *Nr5a1* ablation.

### Loss of SF-1 in Sertoli cells results in testis cord dysgenesis

**Figure 6:**
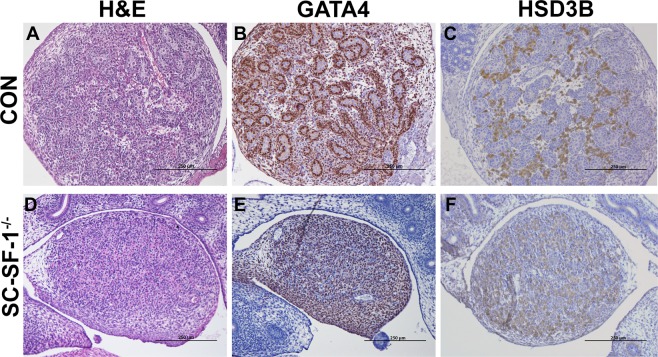
Morphological changes and Sertoli-cell-only phenotype at E18.5 in SC-SF-1^−/−^ testes. Histological analysis of the testis stained with hematoxylin and eosin (H&E) in control (**A**) and SC-SF-1^−/−^ (**D**) mouse embryos at E18.5. H&E staining displayed loss of seminiferous tubules in SC-SF-1^−/−^ testis. Surviving tubules were mostly Sertoli-cell-only (SCO) lacking germ cells. Immunohistochemical staining of GATA4 in control (**B**) and SC-SF-1^−/−^ (**E**) testis. GATA4 staining shows the loss of cord formation in SC-SF-1^−/−^ testes. Immunohistochemistry of HSD3B (Leydig cell marker) in testes from control (**C**) and SC-SF-1^−/−^ (**F**) mice at E18.5. The Leydig cells were found to express HSD3B in SC-SF-1^−/−^ testes suggesting that loss of Sertoli cells did not affect Leydig cell function (scale bar = 250 μm). CON = control.

We analyzed the histology of developing testis to determine the structural defects in the SC-SF-1^−/−^ testes. H&E staining of E18.5 testis revealed that SC-SF-1^−/−^ testes (Fig. 6D) lacked seminiferous cords when compared to the controls (Fig. 6A). Leydig cells were detected using HSD3B staining, and were present between the cords in the control testes and formed well-organized clusters (Fig. 6C). In SC-SF-1^−/−^ testes, Leydig cells expressed HSD3B at levels similar to control (Fig. 6F) and the size of seminal vesicles and epididymis suggest that testosterone generation is maintained at levels similar to that of the control. The areas between Leydig cells seem to be filled with cells that closely resemble fibroblast and may be dedifferentiated peritubular cells (Fig. 6F). Furthermore, using immunoblotting we found HSD3B expression levels to be similar between control and SC-SF-1^−/−^ testes (Supplementary Fig. 7A).

Staining for GATA4 transcription factor, as expected was predominantly expressed in Sertoli cells and Leydig cells of the control testis (Fig. 6B). GATA4 staining demonstrates the loss of cord formation in SC-SF-1^−/−^ testes (Fig. 6E) and the unstained interstitial cells could be remnants of the peritubular cell population released from seminiferous cords. Based on these data, we conclude that deletion of *Nr5a1* within Sertoli cells leads to a loss of Sertoli cells which in turn results in seminiferous tubule degeneration but does not alter Leydig cell numbers (Supplementary Fig. 7B), testosterone biosynthesis function as evidenced by testes descent and adult seminal vesicle weight.

### SC-SF-1^−/−^ male mice exhibit a limited number of functional seminiferous tubules in adulthood while Leydig cell functions persist

**Figure 7:**
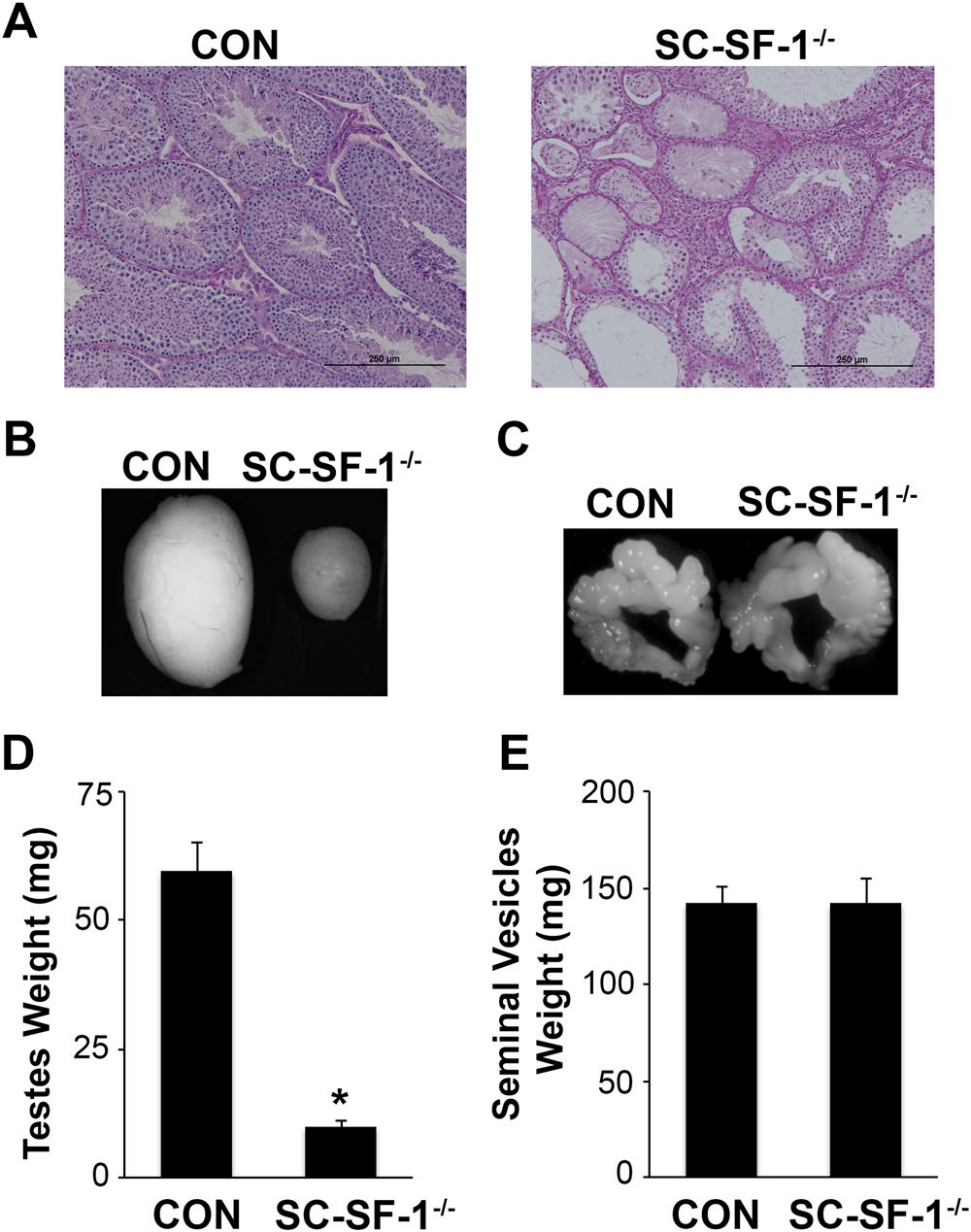
NR5A1 depletion in Sertoli cells impeded testes development. (**A**) Histological analysis of testis sections stained with Periodic Acid-Schiff in control and SC-SF-1^−/−^ mice at 6 weeks of age (scale bar = 250 μm). PAS stain showing abnormal phenotype of the seminiferous tubules including vacuolization and visibly large areas of interstitial cells in SC-SF-1^−/−^ testis. Majority of these seminiferous tubules were devoid of germ cells, forming a Sertoli-cell-only phenotype. Stereomicroscopy images of testes (**B**) and seminal vesicle (**C**) from control and SC-SF-1^−/−^ mice at 6 weeks. Average weight of the testes (**D**) and seminal vesicles (**E**) of control and SC-SF-1^−/−^ mice. The testes were smaller in SC-SF-1^−/−^ mice, while seminal vesicles were similar in size compared to those of control mice. Values are expressed as the mean ± standard error of the mean (SEM) from 5 mice. *p < 0.001 by two-tailed Student’s unpaired *t* test. CON = control.

The loss of testes cords in the developing fetal testis of SC-SF-1^−/−^ mice affected gross testicular morphology in adulthood as expected. The external genitalia of the 6-week-old SC-SF-1^−/−^ males were indistinguishable from their control counterparts. Testes in SC-SF-1^−/−^ mice had fully descended from the inguinal position to the scrotum. We analyzed reproductive parameters (testis, epididymis, vas deferens and seminal vesicles weight) and testis histology. Gross anatomical structures of the adult male knockout consist of very small fully descended testes. The testes of 6-wk-old SC-SF-1^−/−^ males (9.81 mg ± 1.360; N = 4) were significantly smaller (*p* < 0.001) than controls (59.96 mg ± 2.243; N = 4; Fig. 7B,D). Despite the striking difference in testis size, the Wolffian duct derivatives (epididymis, vas deferens and seminal vesicles) were fully developed in SC-SF-1^−/−^ mice. The seminal vesicles were found to be morphologically similar (Fig. 7C) and did not statistically differ in weight (Fig. 7E) between SC-SF-1^−/−^ (141.6 mg ± 13.417; N = 5) and control mice (141.8 mg ± 8.420; N = 5).

Bouin’s fixed paraffin embedded testis sections were PAS stained and evaluated for their internal anatomy. Testis histology was markedly distinguishable between control and SC-SF-1^−/−^ (Fig. 7A) adult testes. Compared to control mice at 6 wk of age, the corresponding sections of testes in SC-SF-1^−/−^ mice showed a very limited number of seminiferous tubules characterized by vacuolization and contained visibly larger areas of interstitial cells between tubules (Fig. 7A). Additionally, many of these tubules in SC-SF-1^−/−^ testes exhibited areas with Sertoli cells and were mostly devoid of germ cells or spermatozoa forming a Sertoli-cell-only phenotype. The small number of Sertoli cells that survived ablation at E18.5 proliferated postnatally and reformed seminiferous cord/tubules many of which lacked germ cells.

## Discussion

The dysgenesis through apoptosis of the gonads post separation of NR5A1 positive adrenal and gonadal primordial cells in the null *Nr5a1* mouse^3,4^ revealed the role of NR5A1 in testicular/gonadal maintenance but did not shed light on its role following gonadal development and sex determination. The current thinking is that Sertoli cells, which start differentiating at E10.5, induce fetal Leydig cell differentiation. At E14.5 Sertoli cells have differentiated to functionally give rise to testis cords that appear as transverse circular loops prior to further convolution at E16.5^31^. In this study, *Nr5a1* for the first time has been ablated specifically in the mouse Sertoli cell at E14.5 (SC-SF-1^−/−^) and illustrates a profound phenotype. By E15.5 testis cords were mostly lost with a concomitant loss of SOX9 and for the first time we have evidence *in vivo* that NR5A1 is required for the maintenance of SOX9 in testes development (Fig. 1). We also see a loss of germ cells at this stage as determined by the loss of VASA (DDX4) staining (Fig. 5). Furthermore, the Sertoli cell population declined by 75% at E15.5 in SC-SF-1^−/−^ mice resulting in the disruption of seminiferous cords with limited cord structure remaining at E18.5 (Fig. 1, Supplementary Fig. 1B) suggesting that NR5A1 is required for Sertoli cell survival during testes development.

*In vivo* mutational studies of the promoter of *Amh* gene strongly suggest a role for *Nr5a1* in the upregulation and maintenance of AMH in the Sertoli cells^32^. Accordingly in Fig. 4, we observed a decline in AMH levels in the fetal testis by E15.5 in SC-SF-1^−/−^. To test if the loss of Sertoli cells was due to a proliferation defect or due to apoptosis, we performed TUNEL and BrdU IHC on E15.5 testes. There was reduced Sertoli cell proliferation in the SC-SF-1^−/−^ E15.5 testes but also considerable apoptosis was observed suggesting testes cord loss may be due to apoptosis, which was confirmed by TUNEL staining analysis performed 12 hours post Cre mediated ablation of *Nr5a1* (Fig. 2). Interestingly, a recent study^33^ revealed that the knockout of *Mdm2* at E14.5 in the Sertoli cell (SC-mdm2^−/−^) using the *Amh-Cre* mouse demonstrated a similar apoptotic phenotype to the SC-SF-1^−/−^. TP53 is a tumor suppressor and *Mdm2*, an oncogene, functions as a ubiquitin E3 ligase ubiquinating TP53 which is then directed through the 26 s proteasome for degradation. MDM2 also directly binds TP53 through it’s amino terminus to block TP53 mediated transcription^34–36^. Thus the loss of MDM2 in the SC-mdm2^−/−^ results in higher levels of TP53, which resulted in increased apoptosis. This result suggests strongly that MDM2 normally suppresses TP53 in the fetal Sertoli cell. The *Mdm2* knockout model led us to examine the MDM2/TP53 pathway as a possible mechanism for apoptosis in the *Nr5a1* knockout mice. MDM2 protein levels were diminished in the E15 SC-SF-1^−/−^ testes (Fig. 3C,D) while TP53 was significantly higher when compared to controls (Fig. 3A,B). We examined the promoters of rat, mouse and human *MDM2* for NR5A1 binding sites. The *MDM2* gene has two promoters, one proximal to Exon1 (P1) and the other in intron 1 (P2)^37,38^. We found NR5A1 binding sites in a highly conserved region in the P1 promoter of mouse *Mdm2* while NR5A1 binding sites are seen in both P1 and P2 promoters in the human (Fig. 3E). Similar to the loss of *Mdm2* in the SC-mdm2^−/−^ model that causes TP53 levels to increase, the loss of NR5A1 regulation of *Mdm2* in SC-SF-1^−/−^ caused the apoptotic phenotype. Although the SC-mdm2^−/−^ male mice showed a higher level of feminization^33^, the secondary feminization phenotype was not evident in SC-SF-1^−/−^ male mice. Figure 6F demonstrates that Leydig cells from E15.5 to E18.5 in SC-SF-1^−/−^ testis are still present and expressing the steroidogenic enzyme HSD3B, a marker for fetal Leydig cells. Furthermore, the levels of HSD3B are similar in SC-SF-1^−/−^ compared to control E15.5 testes (Supplementary Fig. 7A). After E13 Leydig cells only express HSD3B type I at significant levels until postnatal day 10 where HSD3B type VI is expressed^39^. The external genitalia of the adult SC-SF-1^−/−^ males were indistinguishable from their sibling controls. Furthermore, there was no persistence of mullerian derivatives suggesting adequate expression of AMH occurring prior to the complete ablation of *Nr5a1* in the Sertoli cells. On the contrary, the *Amh-Cre* mediated knockout of *Wt1* showed a complete loss of *Amh* by E14.5 and revealed some mullerian persistence^40^ but not as extensively as seen in the SC-mdm2^−/−^. The use of different *Amh-Cre* transgenic mice and the timing of loss of Sertoli cell function may account for these differences. Apoptosis occurs earlier in the SC-Mdm2 ^−/−^ male fetus due to the immediate ablation of *Mdm2* whereas in the SC-SF-1^−/−^ model loss of MDM2 is a delayed consequence of NR5A1 loss. Fetal Leydig cells (FLCs) differentiate and expand between E12.5 and E15.5 and are thought to be induced by differentiating Sertoli cells^41^. The SC-SF-1^−/−^ embryonic testes may be delayed sufficiently to allow for a regular number of FLCs and thus normal masculinization. In the study previously described where Sertoli cell apoptosis was induced by diphtheria toxin (DTX) at postnatal day (PD) 50 and demonstrated that 30 days after injection there was a loss of the Leydig cell population (37% of control) but testosterone levels remained constant. The surviving Leydig cells were hyperactive and produced more testosterone due to higher luteinizing hormone (LH) levels^42^. FLCs survive in conditional knockouts that cause Sertoli cell ablation, as long as it is outside the E12.5 to E15.5 window. In the SC-SF-1^−/−^ we have found that seminal vesicles in 6-wk-old animals show no discernible differences in their structure, size or weight suggesting that androgen action is sufficient for the maintenance of these secondary structures. It is important to note that not all Sertoli cells were ablated and that there were some Sertoli cells that escaped Cre mediated *Nr5a1* ablation. This is not uncommon as Cre mediated deletion can be position dependent and some genes are harder to ablate than others due to local chromatin levels^43^. Recent studies have successfully produced mice with testes devoid of Sertoli cells through Diptheria Toxin (DTX) mediated ablation *in utero* and at birth (pnd2) resulting in a similar adult phenotype as seen in SC-SF1^−/−^ mice. Similar to our study, Rebourcet *et al*. also demonstrated that Sertoli cell numbers could not recover after cell loss during the fetal and neonatal phase^44,45^. The SC-SF1^−/−^ mice had a limited number of seminiferous tubules some of which were Sertoli-cell-only and others had some level of spermatogenesis. By E18.5 there were very few VASA positive cells (Fig. 5) or SOX9 positive cells (Fig. 1) suggesting that some proliferation took place postnatally.

A previous study illustrated the need for five transcription factors (NR5A1, GATA4, DMRT1, SOX9 and WT-1 to reprogram mouse embryonic fibroblasts (MEFs) to induced embryonic Sertoli-like cells (ieSCs)^46^. Genes for four of these five factors have been ablated specifically in the Sertoli cells without an apoptotic phenotype^40,47–49^. The loss of NR5A1, a factor required to reprogram MEFs to ieSCs, causes apoptosis and therefore must play a role as a cell cycle checkpoint in the proliferation and development of the Sertoli cell.

In humans, heterozygous mutations in the *NR5A1* gene were associated with mild to severe sex developmental disorders^50,51^, sex reversal^52^, impaired adrenal development, testicular dysgenesis and female external genitalia^49^. Collectively, these data imply an essential role in the testes for NR5A1 *in utero* for male sexual development. The null and Sertoli cell specific knockout of *Nr5a1* both show an apoptotic phenotype. We for the first time have a tangible model for a mechanism by which the loss of *Nr5a1* causes apoptosis. In future we would like to cross both the null *Nr5a1* and the *Nr5a1*^*fl/fl*^ *Amh*-*Cre* (SC-SF-1^−/−^) mice into a *Tp53* null background to rescue the apoptotic phenotype and study the downstream functions of NR5A1 without the loss of Sertoli cells. Further, the potential interaction of NR5A1 with the MDM2/TP53 pathway *in vivo* will be addressed by these studies.

## Methods

### Generation of mice

All animal protocols were reviewed and approved by the Institutional Animal Care and Use Committee at Wayne State University (Detroit, MI). All experimental methods were carried out in accordance with the guidelines and regulations of the Institutional Animal Care and Use Committee at Wayne State University. Mice were housed in a facility controlled for temperature and humidity with a 12 h light-dark cycle. All mice were obtained from the Jackson laboratory, Bar Harbor, ME. Sertoli cell-specific *Nr5a1* knockout mouse line (*Nr5a1*^*fl/fl*^ *Amh*-*Cre*) was generated by mating *Nr5a1*^*fl/fl*^ (Stock No: 007041, Jackson Laboratories) male mice to *Amh-Cre* (Stock No: 007915, Jackson Laboratories) female mice; the resulting *Nr5a1*^*fl/fl*^ *Amh-Cre* female mice when crossed to *Nr5a1*^*fl/fl*^ male mice deletes exon 7 which encompasses the AF2 (activation function domain 2) of *Nr5a1*. All mice were bred into C57BL/6 J background for at least 5 generations. Food and water were available to animals *ad libitum*.

Timed pregnant mice were obtained by housing male and female mice together between 1800–0600 h. The presence of vaginal plug the following morning was designated as E0.5. Pregnant mice were injected with 5-bromo-2′-deoxyuridine (BRDU; 50 mg/kg b.wt.) intraperitoneally (i.p.) 4 h prior to dissection. Mice were anesthetized with Avertin (2,2,2-Tribromoethanol; 250 mg/kg b.wt., i.p.) and fetal testis tissues from E15, E15.5, E16.5, E17.5 and E18.5 were harvested and immediately fixed in 4% paraformaldehyde or in Bouin’s solution for immunohistochemistry. Additionally, testes were dissected at E15 and 15.5, flash-frozen in liquid N_2_ and stored at -80 °C for immunoblotting. Fetal genotypes were analyzed by PCR using genomic DNA isolated from fetal tail. Primer sequences are as follows: *Nr5a1 lox* forward primer 5′- AGACAAGTGCACCCCATTTC-3′, reverse primer 5′-ACCATCACCAACCGCTAAAC-3′; *Amh-Cre* forward primer 5′-GCATTACCGGTCGATGCAACGAGTG-3′, reverse primer 5′-GAACGCTAGAGCCTGTTTTGCACGTTC-3′. PCR products were resolved by agarose gel electrophoresis and amplicons were determined relative to size markers.

### Immunohistochemistry

Adult mice were transcardially perfused with Phosphate-buffered saline (PBS) followed by 4% paraformaldehyde. Testes were dissected and fixed in 4% paraformaldehyde at 4 °C or in Bouin’s fixative at room temperature (RT) overnight. Post fixation, both the adult and embryonic (E15-E18.5) testes were embedded in paraffin, sectioned at 7 μm thickness, and affixed onto superfrost slides (VWR International LLC, PA, U.S.A.). The slides were air dried and incubated on slide warmer at 60 °C for 20 min and stored at RT. Tissue sections were deparaffinized and rehydrated through a series of xylene/alcohol followed by PBS wash. Antigen retrieval was performed in pressure cooker using citrate buffer (10 mM) with Tween-20 (pH 6.0) for 21 min.

For dual-label IHC for BrdU and SOX9, labeling for BrdU was performed first followed by SOX9. Briefly, sections were sequentially blocked using mouse IgG (PK-2200, Vector Laboratories) for 30 min, 5% normal donkey serum (NDS; diluted in PBS) for 30 min followed by avidin/biotin blocking (SP-2001, Vector Laboratories) for 15 min at RT. Tissue sections were then incubated with mouse anti-BrdU (1:50; 11170376001; Sigma) and rabbit anti-SOX9 (1:200; AB5535; EMD Millipore) overnight at 4 °C. Sections were washed and incubated with biotinylated horse anti-mouse secondary antibody for BrdU (1:500; PK-2200; Vector Laboratories) for 30 min and incubated with Cy3-conjugated streptavidin (2 μg/ml; 016-160-084 Jackson Immunoresearch Laboratories) diluted in PBS for 30 min at RT. Subsequent to BrdU staining, the sections were washed, blocked in 5% NDS for 30 min followed by avidin/biotin blocking for 15 min at RT. Tissue sections were incubated with biotinylated horse anti-rabbit secondary antibody (1:500; BA-1100; Vector Laboratories) for 30 min, followed by Fluorescein-conjugated streptavidin (5 μg/ml; 016-010-084; Jackson Immunoresearch Laboratories) for 30 min at RT. SOX9 and BrdU positive cells in each testis section were counted and analyzed using ImageJ software (https://imagej.nih.gov/ij/docs/pdfs/ImageJ.pdf; National Institutes of Health, Bethesda, Maryland). Briefly, the image was first converted to 8-bit grayscale and the threshold was set to distinguish the cells from background. Automatic cell counting was performed on the binary image. This method has been extensively used for cell counting^53^. We also performed manual counting using Adobe Photoshop CC and found the results to be similar to image J counts. Counting was performed on testis sections for three different male fetuses.

For VASA (DDX4) and AMH dual immunofluorescence, sections were blocked with 1% BSA diluted in PBS for 1 h followed by avidin/biotin blocking and incubated with rabbit anti-DDX4 (1:200, ab13840; Abcam) and goat anti-AMH (1:1000, sc-6886; Santa Cruz Biotechnology) diluted in antibody diluent (S0809; Dako) at 4 °C overnight. VASA was visualized using biotinylated horse anti-rabbit secondary antibody, followed by incubation with Fluorescein-conjugated streptavidin. For AMH visualization, sections were incubated with Rhodamine-conjugated donkey anti-goat (1:1000; 705-295-147; Jackson Immunoresearch Laboratories) for 30 min at RT.

For AMH IHC (Individual IHC at E15.5 and 16.5), sections were blocked in 5% NDS, followed by avidin/biotin blocking and incubated with goat anti-AMH at 4 °C overnight. Sections were washed, incubated with biotinylated donkey anti-goat secondary antibody followed by incubation with Fluorescein-conjugated streptavidin as described above.

All tissue sections were washed and mounted (H-1400, Vector Laboratories). The immunofluorescence was visualized using Olympus IX51 fluorescent microscope with FITC and TXRED filter. Images were captured using Olympus CellSens Standard 1.16 software and processed using Adobe Photoshop CC.

For HSD3B and GATA4 IHC, the endogenous peroxidase activity was quenched by incubation with 3% H_2_O_2_ for 5 min followed by blocking and incubation with anti-HSD3B2 (1:200; ab80500; Abcam) and anti-GATA4 (1:200; ab84593; Abcam) at 4 °C overnight. Sections were washed, incubated with biotinylated horse anti-rabbit secondary antibody (1:500; 30 min) followed by avidin-biotinylated horse-radish peroxidase complex (PK-6100, Vector Labs) for 30 min at RT. Diaminobenzidine was used as a chromogen and sections were counterstained with haematoxylin, rehydrated and mounted (Vectamount, H-5000, Vector labs). Images were captured using Nikon Eclipse 90i microscope. For cell counting, HSD3B stained cells in each testis section were counted using ImageJ software and Adobe Photoshop CC.

### Histology of murine testes

Embryonic (E18.5) and adult (6 wk) testis fixed in Bouin’s solution were sectioned and stained either with hematoxylin and eosin (H&E) or Periodic Acid-Schiff. Images were captured using Nikon Eclipse 90i microscope and processed using Adobe Photoshop CC.

### TUNEL staining

Apoptosis was evaluated in paraffin embedded embryonic testis (E15-E18.5) sections using *In Situ* Cell Death Detection Kit (11684809910, Roche) according to the manufacturer’s protocol. Briefly, tissue sections were deparaffinized through xylene, rehydrated in a series of alcohol and permeabilized with proteinase K (10 μg/mL) for 15 min at 37 °C in a humidified chamber. The sections were rinsed in PBS and incubated with the TUNEL reaction mixture containing terminal deoxynucleotidyl transferase (TdT) and labeling solution (nucleotide mixture fluorescein labelled) for 1 h at 37 °C in a humidified chamber. TUNEL-positive cells were detected using confocal fluorescence microscope (LSM 780, Zeiss) using a fluorescein filter and images were processed using ZEN 2012 software.

### Western blot analysis

Whole cell protein extracts were isolated from E15 and E15.5 testis homogenized in RIPA buffer containing protease and phosphatase inhibitors (Thermo Scientific) using a Bullet Blender (Next Advance, NY). Tissue homogenates were incubated at 4 °C with vigorous shaking for 10 s at 1400 rpm every 5 min on an Eppendorf thermomixer for 30 min and centrifuged at 10000 *g* for 10 min at 4 °C. The supernatant was stored at −80 °C until further use. Protein concentrations were determined using the Pierce BCA Assay Kit. Equal amounts of protein were resolved using NuPAGE precast 4–12% bis-tris gels (Thermo Scientific) and transferred onto Hybond-P polyvinylidene fluoride (PVDF) membrane (Millipore). Following blocking with either in 5% non-fat dry milk or 3% BSA in Tris-buffered saline with 0.1% Tween-20 for 1 h at RT, the membranes were incubated with mouse anti-MDM2 (1:100; sc-965; Santa Cruz), rabbit anti-phospho-p53 (Ser15; 1:400; #9284; Cell Signaling Technology), goat anti-AMH (1:300; sc-6886; Santa Cruz), rabbit anti-SOX9 (1:1000; AB5535; EMD Millipore), anti-HSD3B2 (1:200; ab80500; Abcam) and rabbit anti-Histone H3 (1:1000; #9715; Cell Signaling Technology) overnight at 4 °C. Membranes were washed and incubated with corresponding HRP-conjugated secondary antibodies for 1 h at RT. The immunoreactive bands were visualized using enhanced chemiluminescence detection system (Thermo Scientific). Densitometric analyses of immunoreactive bands were quantified using ImageJ software and normalized to Histone H3.

### Statistical analysis

Differences between groups were analyzed using Student’s unpaired two-tailed *t*-test using GraphPad PRISM version 5.02 (GraphPad Software, Inc., San Diego, CA). Statistical significance was set as a *p* value of < 0.05.

## Supplementary information

Supplementary Figures
