# Supplementary material for: Steroidogenic Factor 1 (Nr5a1) is Required for Sertoli Cell Survival Post Sex Determination
Source: Sci Rep. 2019 Mar 14;9:4452. doi: 10.1038/s41598-019-41051-1 (PMC6418149; doi:10.1038/s41598-019-41051-1)

Steroidogenic Factor 1 (*Nr5a1*) is Required for Sertoli Cell Survival Post Sex Determination.

[1]Prashanth Anamthathmakula[#], [1]Chandra Suma Johnson Miryala[#], [2]Rebecca S. Moreci, [1]Chandrashekara Kyathanahalli, [1]Sonia S. Hassan, [1]Jennifer C. Condon and [1]Pancharatnam Jeyasuria*

[1]Department of Obstetrics and Gynecology, Wayne State University Perinatal Initiative, School of Medicine, Wayne State University, Detroit, MI, USA

[2]Department of Cell Biology, Duke University, Durham, NC 27708

[#]These authors contributed equally to this work.

***Corresponding Author:** Pancharatnam Jeyasuria, Wayne State University, 275 East Hancock Street, C.S. Mott Center #338, Detroit, MI 48201, USA. Tel: (313) 577 2153 Email: suria@med.wayne.edu

Supplementary Figure 1. Cellular localization of NR5A1 in gonad-specific SC-SF-1$^{-/-}$ testes during embryonic development. (A) Immunostaining of NR5A1 in control and SC-SF-1$^{-/-}$ testes at E15.5. NR5A1 which is expressed in both Leydig cells and Sertoli cells is clearly eliminated from the majority of Sertoli cell population as is evident by the loss of cord structures in SC-SF-1$^{-/-}$ testes. The interstitial NR5A1 positive cells are indicative of Leydig cells (scale bar = 200 µm). (B) Number of Sertoli cells relative to control in SC-SF-1$^{-/-}$ testes at E15.5 and E18.5. Utilizing SOX9 as a Sertoli cell marker there was a loss of 75% of Sertoli cells by E15.5 and this increases to 81% by E18.5 in the SC-SF-1$^{-/-}$ testes compared to their respective controls. CON = control.

Supplementary Figure 2. NR5A1 ablation in Sertoli cells led to progressive decline in germ cell population. Double immunostaining for AMH (red) and VASA (green) was performed in testes at E16.5 and E17.5 from control and SC-SF-1$^{-/-}$ embryos. Sertoli cells (labeled by AMH) and germ cells (labeled by VASA) depict a normal cord-like structure in the control testes at E16.5 (A) and E17.5 (B). A pronounced loss of AMH and VASA staining was observed in SC-SF-1$^{-/-}$ testes suggesting a marked decline in Sertoli cell and germ cell population at E16.5 (C) and E17.5 (D). CON = control. Scale bar = 200 µm.

Supplementary Figure 3. ClustalW multiple sequence alignment of Rat and Mouse *Mdmd2*. Sequence alignment showing NR5A1 response element (AGAGGTCA) in a highly conserved region in the P1 promoter of both rat (-1089 position) and mouse (-776 position) *Mdm2*.

Supplementary Figure 4. Reduced expression of SOX9 in SC-SF-1$^{-/-}$ mice. SOX9 protein levels were analyzed by western blotting in whole tissue extracts of control and SC-SF-1$^{-/-}$ mice at E15.5. Histone H3 served as a protein loading control. CON = control.

Supplementary Figure 5. Expression of phospho-TP53 (p-TP53) and MDM2 in SC-SF-1$^{-/-}$ mice. (A) The activation of TP53 was investigated in testis of SC-SF-1$^{-/-}$ mice. The expression of p-TP53 levels was higher in SC-SF-1$^{-/-}$ testes. (B) MDM2 showing a decrease in the protein level in SC-SF-1$^{-/-}$ testes compared to control. Histone H3 served as a protein loading control. CON = control.

Supplementary Figure 6. Loss of NR5A1 in Sertoli cells decreases AMH expression. AMH protein levels were analyzed by western blotting in whole tissue extracts of control and SC-SF-1$^{-/-}$ mice at E15.5. Histone H3 served as a protein loading control. CON = control.

Supplementary Figure 7. HSD3B expression in control and SC-SF-1$^{-/-}$ testes. (A) Immunoblotting of HSD3B (Leydig cell marker) in E15.5 testes from control and SC-SF-1$^{-/-}$ mice revealed similar HSD3B expression levels. Histone H3 served as a protein loading control. (B) Number of Leydig cells relative to control in SC-SF-1$^{-/-}$ testes at E18.5 utilizing HSD3B as a Leydig cell marker. CON = control.

Supplementary Figure 1

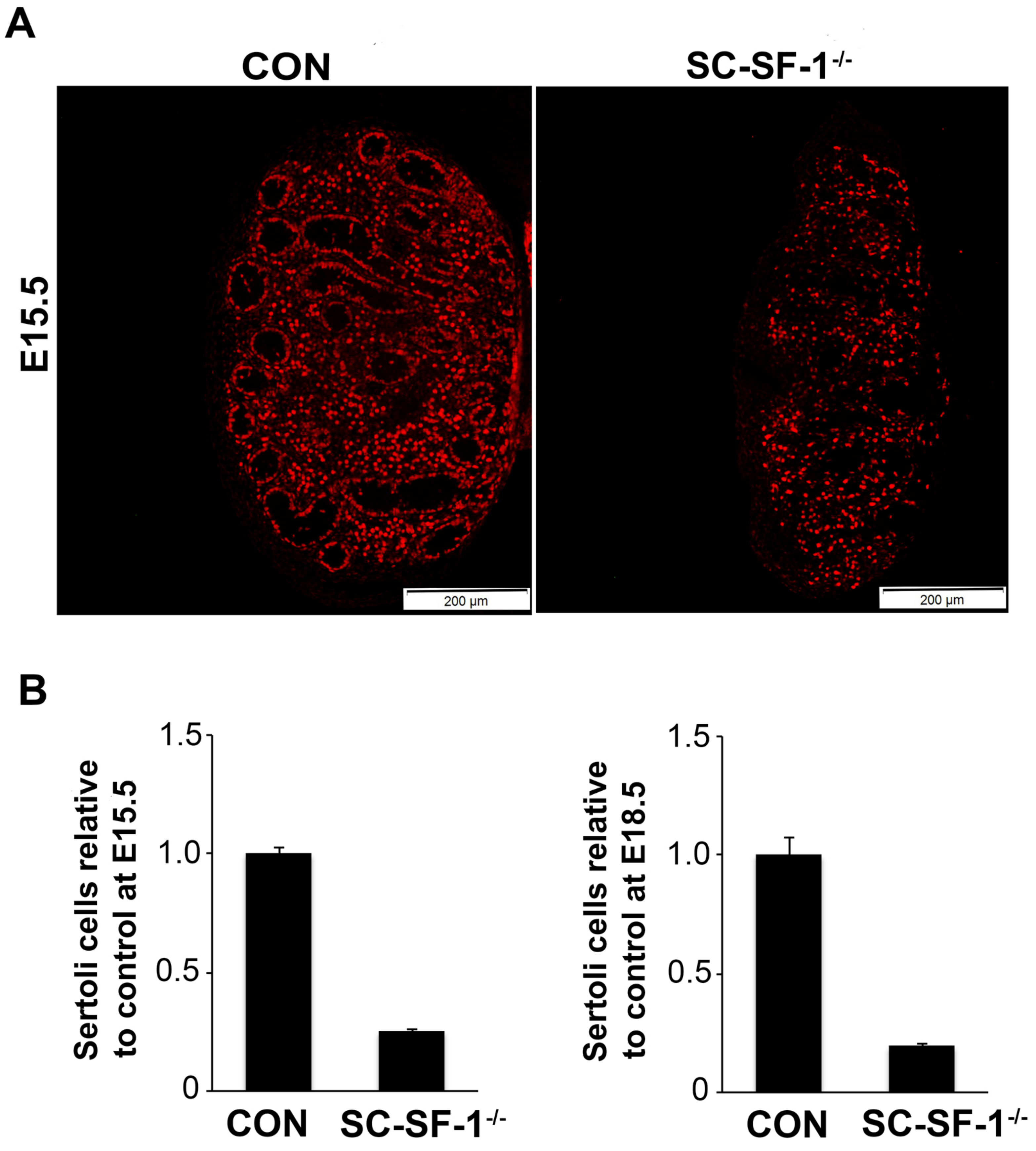

Supplementary Figure 2

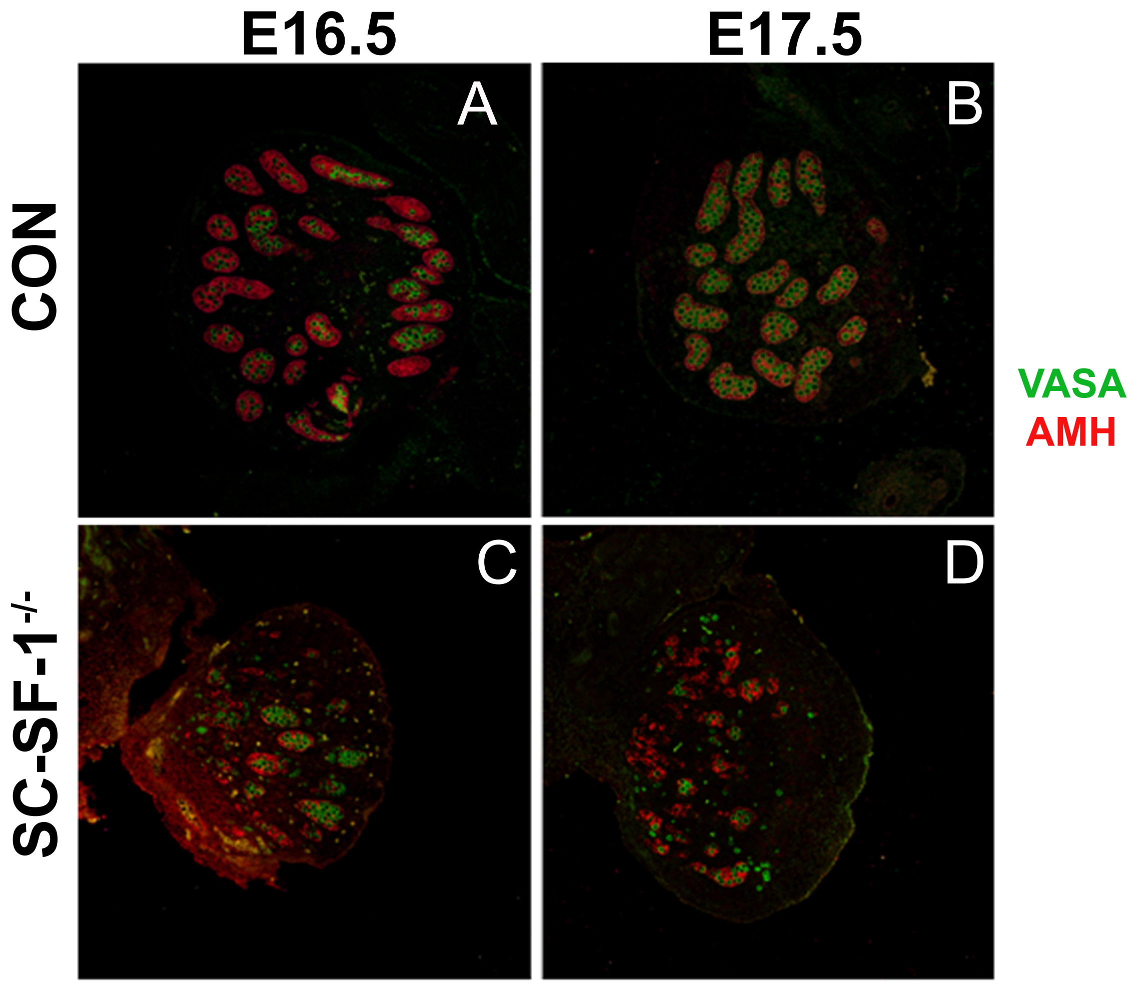

Supplementary Figure 3: ClustalW multiple sequence alignment of Rat and Mouse Mdmd2 gene. Sequence alignment showing SF-1 response element (AGAGGTCA) in a highly conserved region in the P1 promoter of both rat (-1089 position) and mouse (-776 position) Mdm2 gene.

```
ClustalW multiple sequence alignment

2 Sequences Aligned           Processing time: 0.7 seconds
Gaps Inserted = 26            Conserved Identities = 963
Score = 967

Pairwise Alignment Mode: Slow
Pairwise Alignment Parameters:
    Open Gap Penalty = 25.0   Extend Gap Penalty = 6.7

Multiple Alignment Parameters:
    Open Gap Penalty = 15.0   Extend Gap Penalty = 6.7
    Delay Divergent = 30%     Transitions: Weighted

Rat mdm2     1                                                                 0
Mus mdm2     1 GAACTGTTGGGGAAATGAAGGCTGTCAGTAAGAAGTGGCAAGCCCCAAAGAACGAAATTA   60


Rat mdm2     1                                                                 0
Mus mdm2    61 CCTTTCTCCTTTAGGTAGGTTATCAATGTTACTCAAATTAGCTTTAAATTGCTCAACTCA  120


Rat mdm2     1                                                                 0
Mus mdm2   121 AGCTATCCTCCTGCCTCAGCTTCCTGATTATCTTTGACTGTGCAAATGTGCCACCACCCC  180


Rat mdm2     1                                                                 0
Mus mdm2   181 TGGTTTTAAGTCCTTTAAGATCTGGGGCTTTTGCTTTGGGGACCGAGGACTGAACAATGC  240


Rat mdm2     1                                                                 0
Mus mdm2   241 AGGACCTTCTCGGTGCCACACACCCTCCGTTCTTAGATGTACCTTAAAAGGTGGACCCAG  300


Rat mdm2     1                        ATTACAGACGTGCAGCATTACATCTGGCCTTGTCA-T   36
Mus mdm2   301 ACGGCTGTTGCTCTGTACCTGGGATTACA--CGCACACCATTATATCTGGCCTTATCAAT  358
                                      ******  **  ** ***** ********** *** *

Rat mdm2    37 GCTTTTCTCCCTTTTACCTGTGTCCACACCACAGAAAGCAGCAGCTTCAAGGTCCAATTC   96
Mus mdm2   359 GCTTTT----CTTTGACCTGCGTCCACACTGCCTAAAGCAGCG---TCCAGGCCAAACTC  411
               ******    **** ***** ********  *  ********    ** *** * ** **

Rat mdm2    97 CTATAACTTAGAATGCTATATTGGGCCCTCATGATAACCTCCAGTTAGAAAGTTTGTAAA  156
Mus mdm2   412 CTATACCTTATAATGCTATCTTGGGCCCTCAAGATGACTAACAATTAGAAAGTTTGTAAA  471
               ***** **** ******** *********** *** **   ** ****************

Rat mdm2   157 CACTGGAGTGTATGTTTTGAGGAGGC-AGGCTGATTCCATGACAAGCTTCAGATTGGCAG  215
Mus mdm2   472 CACTGGAGTGTATGTGTTGAAAAGGCCAGGCTGATTTCATGACAAGTTTCAAACTGGCAG  531
               *************** ****  ****  ********* ********* **** * ******

Rat mdm2   216 TTAAGGAGAGTAGGCCTAAATCTCTGTTTCCAAGAACTGGGCAATTCCAGGCAAGTCCAG  275
Mus mdm2   532 TTAAGGGGACTAGGCCTAAATCTCTGTTTCCAAGAACTGGGCAATTCCGGGCAAGTCCGG  591
               ****** ** ************************************** ********* *

Rat mdm2   276 GCCTCAAAAGCTGCGCAGGGCCTGGCCTGAGGCAAGGATAATGGGTCAGCAGCAGTTTCC  335
Mus mdm2   592 GCCTCAGAAGCTGC-------CCCGCCTGAGGCAAGGACAATGGGTCAGCAGCAGTTTCC  644
               ****** *******       *  ************** *********************

Rat mdm2   336 AGACCTCC----CCACAAGTTTCCAAGCTCCTCACCCCAGCAATGGAATGTCCACAGAGA  391
Mus mdm2   645 AGACTTTCTCAGCCAAAAGTTTCCAGCCCTCTCACCCCGGGAATGGAATGTCCCTAAAGA  704
               **** * *    *** *********  *  ******** * ************  * ***
                                    Sf1-RE
                                   RRAGGTCA
Rat mdm2   392 TGGACTCAACAGATTAACACAGAGGTCACCTACCCCAGAATACCCTAATGTGCTTTAAAT  451
Mus mdm2   705 TGGACCCAATAGATTAACATAGAGGTCACCTATCCCAGAATTCGCTAATATGCTTTATGT  764
               ***** *** ********* ************ ******** * ***** *******  *

Rat mdm2    452 CAGGCCTGCAAGCTCACTTGGTTGCCT--ATCTTAGTAATGGGGAGA-----CCCCAGTA  504
Mus mdm2   765 CAGGTCTGCAAACTCACTTGGGTGTCTCTATCTTAGTAATGGGGAGAGGAGACACCAGAA  824
```

```
               **** ****** ********* ** **  ******************     * **** *

Rat mdm2   505 TGCTGGATTTCTGCAGAATAAAACTCTTTTTTTTTTTTTTTTTTTTTGGTTCTTTTTCTC  564
Mus mdm2   825 TGCTG-ACTTCTGCAGATTAAAAC------------------------------------  847
               ***** * ********* ******

Rat mdm2   565 GGAGCTGGGGACCGAACCCAGGGCCTTGCGCTTCCTAGGTAAGCGCTCTACCACTGAGCT  624
Mus mdm2   848 ------------------------------------------------------------  847


Rat mdm2   625 AAATCCCCAGCCCCAGAATAAAACTCTTTGTGTTTACATACTATGAGTTCAGGGTGTCAT  684
Mus mdm2   848 ----------------------ATTCTTTGCGTTTACATACTATGAGCCCAAGGTGTCAT  885
                                     * ****** ****************  ** ********

Rat mdm2   685 GGACCCTTATCAGCTCAATAGCTTTTAAGTTCTTTTAACTTAAATTTTTTTTCTTTTTTT  744
Mus mdm2   886 GGACCCT---------AACAGTCTTTGAATT-----------------------------  907
               *******         ** **  *** * **

Rat mdm2   745 CTTTTTTTTTTTTTCGGAGCTGGGGACCGAACCCAGGGCCTTGCGCTTTCTAGGCAAGCG  804
Mus mdm2   908 -TTTTTTTTTAATT----------------------------------------------  920
                *********  **

Rat mdm2   805 CTCTACCACTGAGCTAAATCCCCAACCCCTTAACTTAAATTTTAATTATTTCTGTACACA  864
Mus mdm2   921 -----------------------------TTAACTTTAATTATGTGTATGTCTGTGCACA  951
                                            ******* **** *   *** ***** ****

Rat mdm2   865 TAGAGTGCAGTTGCCCATGGAAACCAGAGGAGTACGTTAGGTCCCCTGGAATGACAGGGT  924
Mus mdm2   952 CAG-GTGCGGTTGCTCATGGAGACCAGAAGAGTGTGTTGAGTACCCTGCAATGACAGAAT 1010
                ** **** ***** ****** ****** ****  ***  ** ***** ********  *

Rat mdm2   925 TTGTGTGGCGTGGGTGATTGAAAACCTAAAAAGGGAGCAGTATGCATTTTTTACCACGGA  984
Mus mdm2  1011 TTGTGTGGCGTGAGTGACTGAAAACCTAAAAAGG-AGCTGTATGCACTCTTGACCATGGA 1069
               ************ **** **************** *** ******* * ** **** ***

Rat mdm2   985 ACCATCTCCTCAGTCCTATTTTTATTATTTTTTTATTCACTTTACATCCCGCTCTCTGTC 1044
Mus mdm2  1070 GCTATTC-----ATTTTTTATTCATTATTATTT-ATTCACTTTCCACCCCGCTCTCCGTT 1123
                * **        *  * * ** ****** *** ********* ** ********* **

Rat mdm2  1045 CCCTTCCAGGTCAGCCT-TTCCCACAATCCTTCCCCCCTCCCCGCCTCTAAGGGAGTGGG 1103
Mus mdm2  1124 CCCTTCTGGGACACCCCCTTCTCACAATTTCCTCCCCGTCCC-----------------A 1166
               ******  ** ** **  *** ******     **** ****

Rat mdm2  1104 CTCCAGCCCAAATCCGTACCCCCACATTGCCCATTTTTAGTTTAAAGTCTGAAAATAACT 1163
Mus mdm2  1167 CTCCACCCT---------CTCTCACCTTGCCTATTTTTAAAGTCTTAAAAAAAAATCACT 1217
               ***** **          * * *** ***** *******   *        ***** ***

Rat mdm2  1164 CATTTGTATACAACTTCAGCTGCTGGCGGGATGGGTTTAAAAATACTACCCAGCTGTTAC 1223
Mus mdm2  1218 CATTTGTATACAACTTCAGCTGCTGGCGGGATGGATTTAAAAATACTACCCAGCTGTTAG 1277
               ********************************** ************************

Rat mdm2  1224 TCTTGCTTCCGCTTTGGTAGCAGCAACAGTTGGATTTGAATTTGCCTAGTTACCATTGCG 1283
Mus mdm2  1278 TCTTGCTTTTCTTTTGGTAGCAGCAGCAGTCGGATAGGAACTTGCCTAGCGACCATTGCG 1337
               ********    ************* **** ****  *** ********  *********

Rat mdm2  1284 GTTT-GAGTG-TAAACACAACAGAGCAGCGCGGTACCGCGACCCCTCCCCACTTCCTCCT 1341
Mus mdm2  1338 GTTTCGAGCGGTAAACACAACCG-------------CGCGGCCCCTCCCCACCTCCTGCG 1384
               **** *** * ********** *             **** *********** **** *

Rat mdm2  1342 CGCTCAAGCACGGCGCGCCCACTGAGCCCCGCCCAGGGCCCGCCCCCATCCCCTATTGGT 1401
Mus mdm2  1385 CGCTCCGGCACCGGGCGGCCGCTAAGCCCCGC----------CCCCCGCCTCCTATTGGT 1434
               *****  **** * *** ** ** ********          *****  * *********

Rat mdm2  1402 TCAGGAGGCGGTGCCGGGCGCGCGCGCTCG--------GATGCCCGGATGGCCCTTGGCT 1453
Mus mdm2  1435 CCAGGAGGCGGCGTCGGGCGTGCGCGCGCGCACGCCCCGATGCCCGGATGGCCCGTGGCT 1494
                ********** * ****** ****** **        **************** *****

Rat mdm2  1454 TCCGGTGCTCTGCCCGCGGACCATAGAGGGCGCTCGTCACGGAACTC 1500
Mus mdm2  1495 TCCGGC                                          1500
               *****
```

Supplementary Figure 4

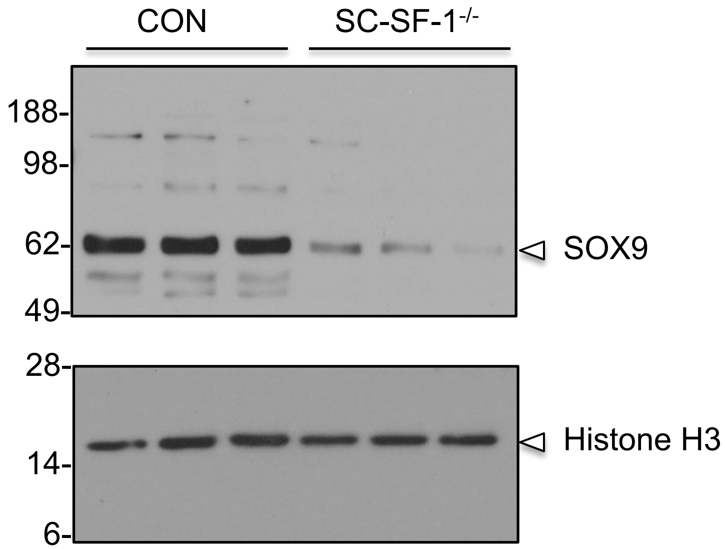

These two blots were cut from one membrane and are the uncropped images of the blots in Figure 1M. The region from 49 kDa to 28 kDa of this blot was used to probe HSD3B and is depicted in Supplementary Figure 7.

Supplementary Figure 5

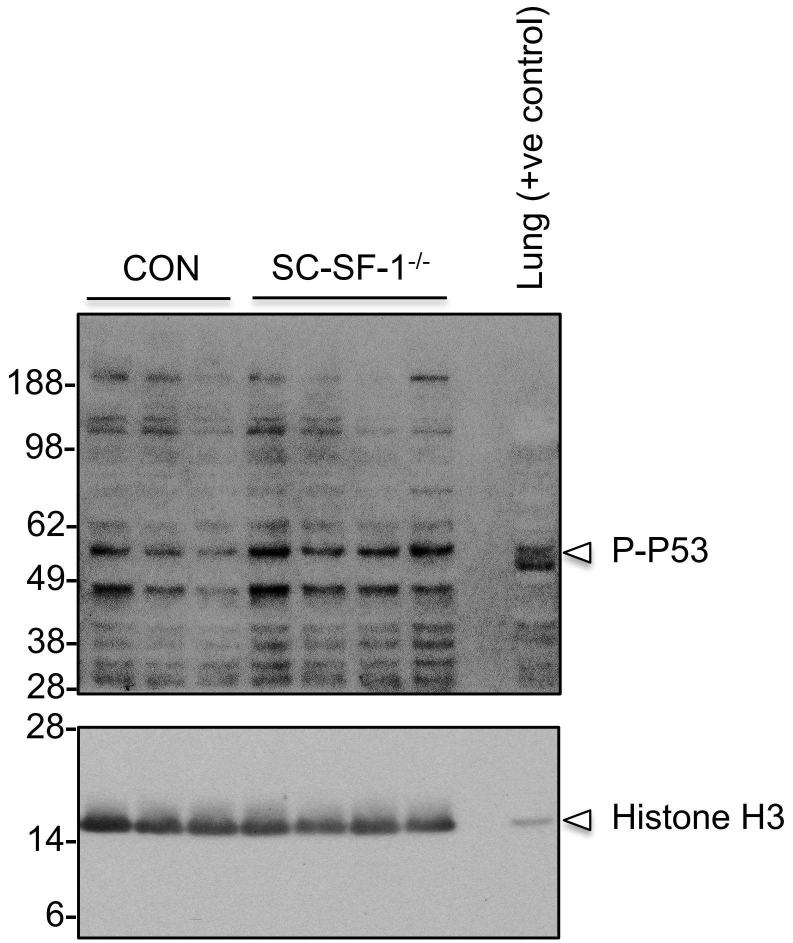

These two blots were cut from one membrane and are the uncropped images of the blots in Figure 3A.

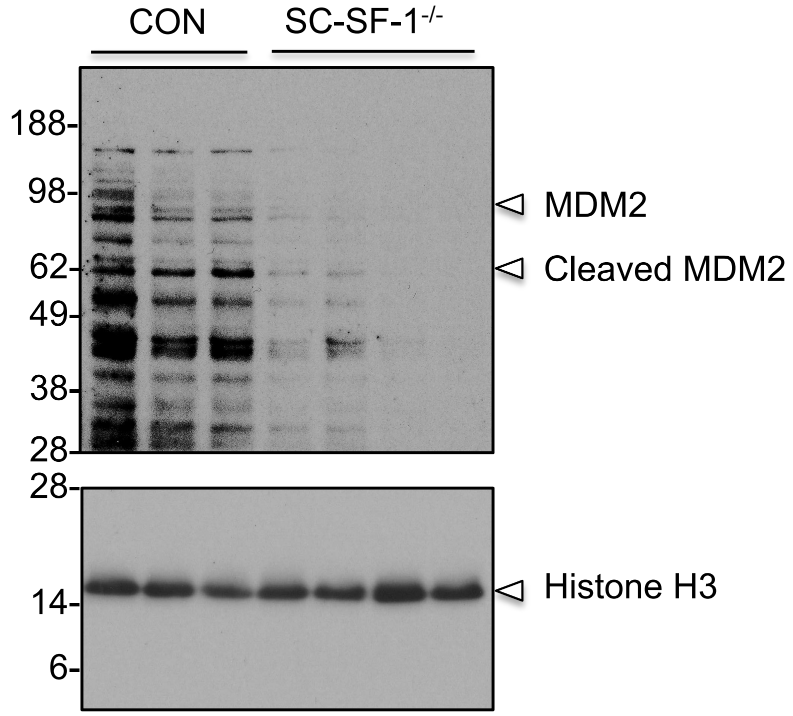

These two blots were cut from one membrane and are the uncropped images of the blots in Figure 3C.

Supplementary Figure 6

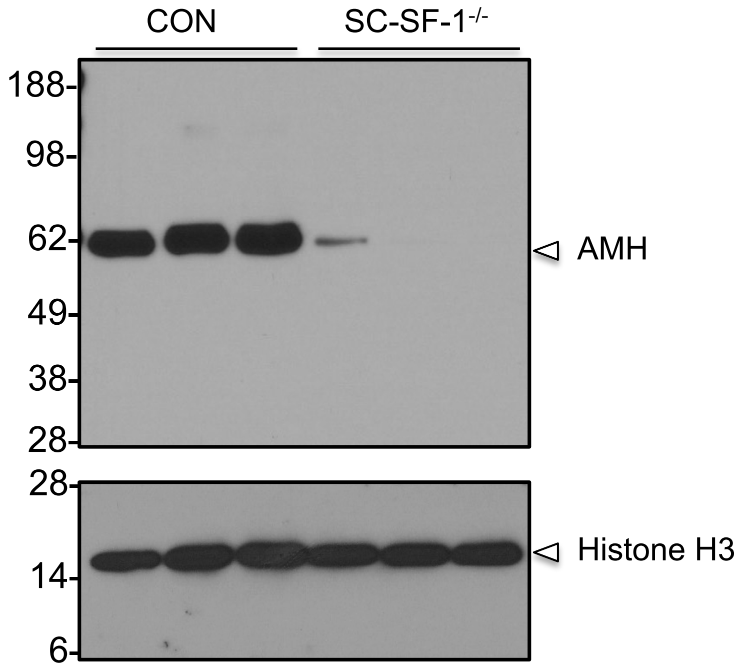

These two blots were cut from one membrane and are the uncropped images of the blots in Figure 4B.

Supplementary Figure 7

A

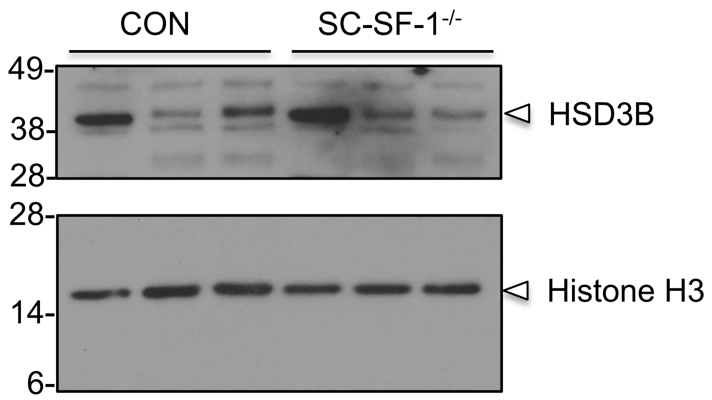

These two blots were cut from one membrane and were probed for HSD3B and Histone H3. The region from 188 kDa to 49 kDa of this blot was used to probe SOX9 and is depicted in Figure 1M and Supplementary Figure 4.

B

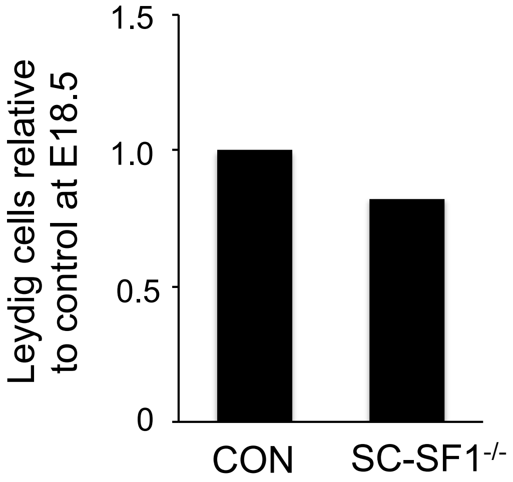

Supplement: Supplementary file 1 — Supplementary Figures [file 41598_2019_41051_MOESM1_ESM.pdf]
